# Neuropathological changes in the TASTPM mouse model of Alzheimer’s disease and their relation to hyperexcitability and cortical spreading depolarization

**DOI:** 10.1038/s41598-024-57868-4

**Published:** 2024-03-27

**Authors:** Fátima Gimeno-Ferrer, Annett Eitner, Noor Noora, Reinhard Bauer, Christoph Schmidt-Hieber, Hans-Georg Schaible, Frank Richter

**Affiliations:** 1 Institute of Physiology 1/Neurophysiology, Jena University Hospital, 07740 Jena, Germany; 2 Department of Trauma, Hand and Reconstructive Surgery, Experimental Trauma Surgery, Jena University Hospital, 07740 Jena, Germany; 3 Institute of Molecular Cell Biology, CMB-Center for Molecular Biomedicine, Jena University Hospital, 07740 Jena, Germany

**Keywords:** Neuroscience, Physiology, Neurology

## Abstract

Although Alzheimer’s disease (AD) is characterized by distinct pathological changes, their precise impact on cortical functions are not well understood. Here we used TASTPM mice as an AD model and asked whether the development of neurodegenerative changes has an impact on the extracellular space (ECS) and neuronal excitability, in particular cortical spreading depolarization (CSD) which requires intact neuron and glial functions. We studied wildtype (WT) and TASTPM mice (3, 6, and 12 months old). TASTPM mice showed progressive proliferation of neocortical Amyloid-beta (Aβ) plaques between 3 and 12 months (more deposits in females than in males) and Aβ accumulation in cortical vessels. As plaques proliferated, neuroinflammatory microglial reaction (CD68, CD39 and Galectin-3) and astrogliosis (GFAP) developed progressively. The cortical ECS volume shrank significantly to about half the size of the WT. CSD in both WT and TASTPM mice showed considerable heterogeneity but did not correlate with the histological changes. However, CSDs were easier to elicit in TASTPM than in WT mice at 3 months, and also compared to older TASTPM mice. Moreover, TASTPM mice showed more hyperexcitability manifested as clonic-tonic behavior after sodium thiopental anesthesia. Thus, AD pathology was associated with abnormal hyperexcitability but did not homogenously alter CSD susceptibility.

## Introduction

Homeostasis refers to the set of autoregulatory mechanisms that maintain the consistency in the composition and properties of an organism’s internal medium. This regulation is essential in the central nervous system (CNS), to counteract pathological challenges^1^ including neurodegeneration. Neurodegenerative conditions are characterized by the development of neuroinflammatory features consistent with aberrant homeostasis. Alzheimer’s disease (AD) is the leading cause of neurodegeneration and dementia (60–70% of dementia cases), affecting more than 50 million individuals worldwide^2^. It is clinically characterized by memory impairments, language problems, disorientation, mood swings, loss of motivation, self-neglect, and behavioral alterations^3^. The diagnosis of AD is confirmed by histological identification of protein inclusion aggregates mainly in hippocampus and cortex. AD is characterized by the progressive development of extracellular Amyloid-beta (Aβ) plaques throughout the brain, especially in the cortex and hippocampus, and the formation of intracellular phosphorylated-Tau (P-TAU) protein. In addition, an activation of glial cells has been described^4^. While AD has substantial impact on structural features, electrophysiological functions and parameters and their relationship to histopathological changes in AD cortices in vivo are poorly understood.

A parameter used to characterize cortical function is the cortical spreading depolarization (CSD), which is an en masse depolarization of neurons and glial cells requiring intact ion gradients and synaptic transmission^5^. CSD forms the neurophysiological basis of the migraine aura^6^. Moreover, CSDs occur in damaged brain tissue following stroke or brain trauma, where they disrupt ion gradients and water distribution, thereby further altering homeostasis in the affected brain area^7^. In the healthy brain of rodents CSDs can be consistently induced by KCl microinjection. Changes of typical CSD parameters (amplitude, propagation velocity, ignition threshold) reveal changes of cortical homeostasis and excitability. Such changes were consistently observed after topical application of neuropeptides or cytokines on cortex which induced neuroinflammatory alterations^8,9^.

In this work, we aimed to characterize the interplay between the progression of AD in the mouse cortex, alterations in cortical homeostasis, and possible changes of CSDs with neurodegeneration. We chose the TASTPM model of AD, which harbors mutations in the *App* and *Psen1* genes that lead to deposition of Aβ plaques. Histopathological characterization of AD (plaque development, glial response) was performed in TASTPM cortices 3, 6 and 12 months of age and in age- and sex-matched controls. Since progressive neuronal loss and glial activation have been extensively described in AD^10^ and functional neurons and glial cells are necessary for CSDs^11^, we expected significant changes in the occurrence and shape of CSDs recorded in male and female WT and TASTPM animals at 3, 6 and 12 months of age. We also monitored electroencephalogram (EEG), and performed extracellular space (ECS) volume measurements.

## Materials and methods

This study was approved by the Thuringian Government (Thüringer Landesamt für Verbraucherschutz) under approval number UKJ-19-021 and was conducted in accordance with the German Animal Welfare Act. All animal studies were ethically reviewed and conducted in accordance with European Directive 2010/63/EEC. Data collection, analysis, and presentation are in accordance with the ARRIVE guidelines. Supplementary Tables 1 and 2 summarize the types of experiments carried out in this study, and the numbers of mice in each experimental group.

### TASTPM mouse model of Alzheimer’s disease

Experiments were performed on wildtype (WT) C57BL/6N animals and AD animals. Adult males and females were divided into age groups (3, 6 and 12 months) (maximum 10 animals per group). The AD model used consisted of heterozygous double-mutant TASTPM transgenic mice obtained from GSK^12^. The TASTPM strain was generated using TAS transgenic mice expressing the Swedish mutant human APP (695-aa isoform) under the control of the murine Thy-1 promoter (Thy-1.APPswe, abbreviated as TAS10) and transgenic TPM mice overexpressing the PSEN1 M146V mutation driven by the murine Thy-1 promoter (Thy-1.PSEN-1.M146V, abbreviated as TPM)^13,14^. Homozygous sperm double-mutant for TAS10 and TPM (homozygous TASTPM) was kindly provided by GSK, UK. This sperm was used to inseminate C57BL/6N WT females to produce a heterozygous TASTPM double-mutant for breeding. All animals were housed in the animal facility at Jena University Hospital. They were maintained on a 12 h day/night cycle with ad libitum access to food and water.

### DNA extraction from ear biopsies

Mouse genomic DNA (gDNA) was extracted from ear punch samples taken at the time of animal identification. Biopsies were incubated overnight at 55 °C and 500 rpm with 300 µL lysis buffer (composition in mmol/L: NaCl 50, Tris–HCl 25 pH 8, SDS 0.17, EDTA 5) and 30 µL of proteinase K (1 mg/ml) (Merck, Darmstadt, Germany). The extracted material was precipitated by centrifugation at 13,000 rpm for 10 min. The gDNA pellet was then precipitated by adding 500 µL 2-isopropanol, mixing and centrifuging for 20 min at 13,000 rpm at 4 °C. The DNA pellet was then washed with 70% ethanol (v/v) by centrifugation for 10 min at 13,000 rpm at 4 °C. Finally, gDNA was resuspended with 200 µL of sterile water (Ampuwa^®^, Fresenius Kabi Deutschland GmbH, Bad Homburg vor der Höhe, Germany) and incubated for 10 min at 60 °C at 500 rpm. The final concentration of gDNA was approximately 10 ng/µL.

### Genotype characterization of the mice

The genotype of each animal resulting in a litter was characterized by polymerase change reaction (PCR) to distinguish between WT, TAS, TPM and TASTPM. PCRs were performed using the MyCycler thermocycler (Bio-Rad, Hercules, CA, USA) according to the manufacturer’s recommendations. The primers TAS-Forward (CAGCTGGTTGACCTG TAGCTTT) and TAS-Reverse (GTGTGCCAGTGAGATGA) were used to identify the TAS10 mutation. For TPM mutation genotyping, the TPM-Forward (CAGCTGGTTGACCTGTAGCTTT) and TPM-Reverse (ATGCTTGGCGCCATATT TCAATG) primers were used. Primers were synthesized by Metabion International AG (Planegg, Germany). For PCR genotyping, a mix was prepared for each mutation. The PCR mix for each mutation contained 15 µL sterile water (Ampuwa^®^, Fresenius Kabi Deutschland GmbH), 2.5 µL Taq Polymerase Buffer (10 × ThermoPol^®^ Reaction Buffer B9004S, New England BioLabs, Ipswich, MA, USA), 1.5 µL MgCl_2_ (25 mM MgCl_2_ Solution B9021S New England BioLabs), 15 1 µL dNTPs (dNTPs Mix 10 mM each R0192 Thermo Scientific, Waltham, MA, USA), 1 µL primer forward (10 pmol/µL, final dilution for PCR 0.4 pmol/µL), 1 µL primer reverse (10 pmol/µL, final dilution for PCR 0.4 pmol/µL), 0.5 µL Taq DNA Polymerase (Taq DNA polymerase M0267S 5000 U/ml, New England BioLabs, Ipswich, MA, USA) and 2.5 µL of gDNA. The PCR conditions were as follows: initial denaturation at 95 °C for 10 min followed by 35 cycles of 95 °C for 15 s (denaturation), 58 °C for 15 s (annealing) and 72 °C for 30 s (elongation). After PCR, DNA fragments were visualized on a 1% agarose gel in 1 × TAE buffer (Tris 40 mmol/L, acetate 40 mmol/L, EDTA 1 mmol/L, pH 8.0) as described^15^ and containing a 1/20,000 dilution of SYBR Green (SYBR green I nucleic acid gel stain #S57563, Invitrogen by Thermo Scientific, San Diego, CA, USA). PCR product samples were diluted with 1/6 loading buffer (Green Gel Loading Buffer 6 × PCR-250-GR, Jena Bioscience, Jena, Germany). To compare the PCR product of the samples, we added a line marker (GelPilot Mid-Range Ladder #239135 Qiagen, Hilden, Germany) to orient the PCR product size. Electrophoresis was performed at a continuous voltage (110 V) and current intensity (220 mA) for 90 min. The final visualization of the DNA bands was obtained using UV illumination and a CCD camera system (Synoptics, Dresden, Germany). The fragments obtained by PCR have a size of 500 bp in the presence of the TAS10 mutation and a size of 350 bp in the presence of the TPM mutation. The absence of these mutations is indicated by the absence of bands. This system does not allow distinguishing between homozygous and heterozygous state of the mutations. However, the breeding was programmed to obtain only mutations in a heterozygous state.

### Anesthesia and surgical preparation of the mice

**Figure 1:**
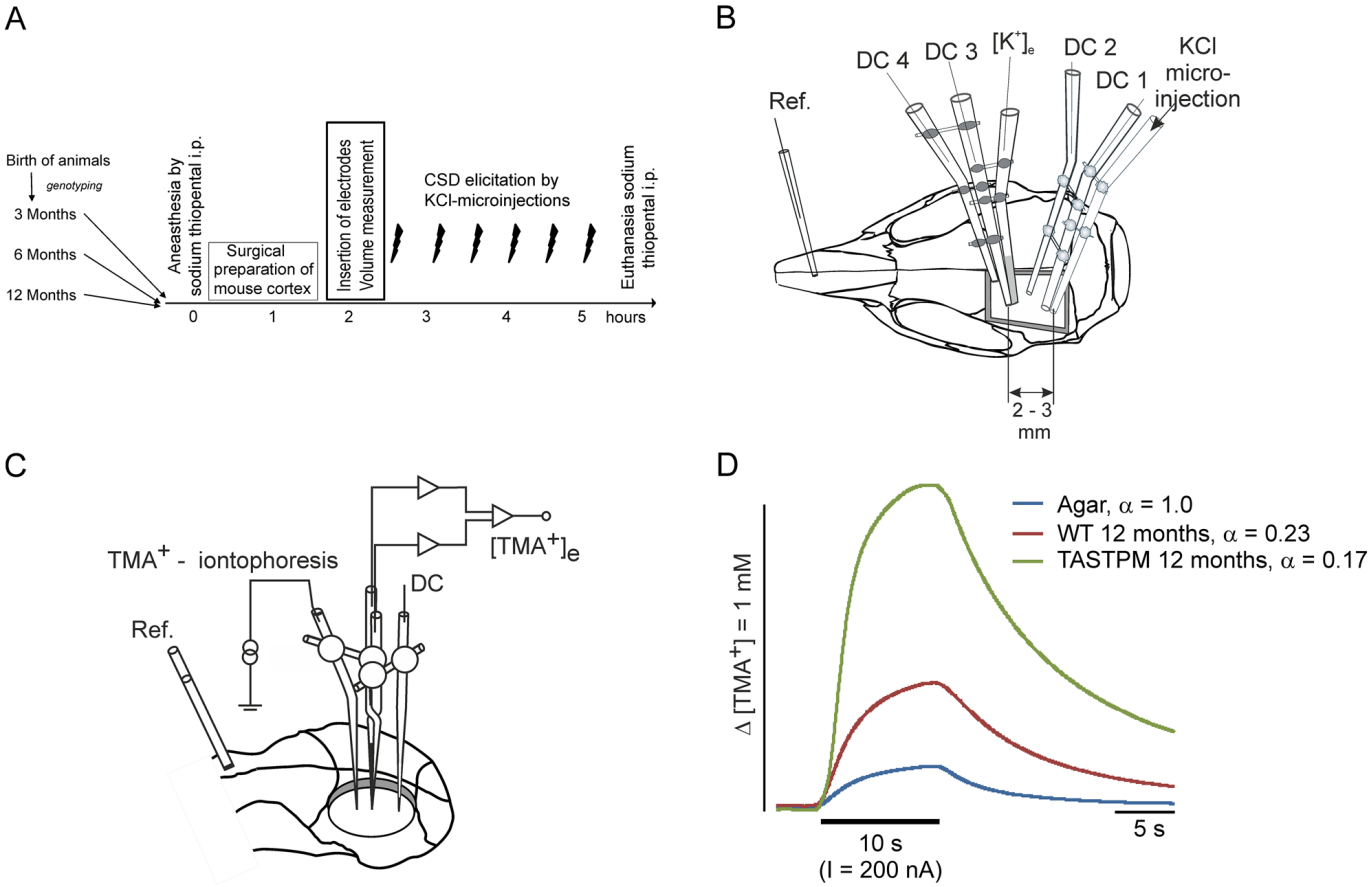
Electrophysiological setup for this study. (**A**) Schedule of an experiment with recordings of ECS and subsequent elicitation of CSD by KCl. Arrows on the left symbolize breeding and daily observation of the mice in the animal facility. (**B**) Schematic drawing of the mouse skull (not to scale) with a trephination surrounded by dental acrylic, the reference electrode on the nasal bone and the set of electrodes for CSD recordings at a depth of 200–250 µm (cortical layer III). (**C**) Schematic drawing of the mouse skull (not to scale) and the TMA^+^ iontophoresis electrodes. (**D**) Typical examples of TMA^+^ recordings in agar gel, male WT cortex and male TASTPM cortex with corresponding values of ECS volume fraction (α).

Adult female and male C57BL/6 WT and heterozygous double-mutant TASTPM mice (n = 83; 20–35 g, aged 3, 6 and 12 months old, housed in the animal facility of Jena University Hospital) were deeply anesthetized with sodium thiopental (Trapanal^®^; Inresa, Freiburg, Germany; initially 100–125 mg/kg intraperitoneally (i.p.)). Mice used for histology were perfused directly after the animals were deeply anesthetized. Mice used for electrophysiology were maintained under anesthesia until the end of the experiment (approximately 5–6 h, see Fig. 1A). During surgery, the depth of anesthesia was regularly monitored by checking the absence of reflexes (corneal blink and reflexes to noxious squeezing of the interdigital skin). During the experiments, if necessary, supplemental doses of sodium thiopental (doses 20 mg/kg) were administered i.p. if necessary to maintain the depth of anesthesia. The trachea was cannulated to ensure spontaneous respiration. A butterfly needle with syringe was placed i.p. to administer any compound if necessary. The electrocardiogram was continuously monitored, and body temperature was maintained at 37 °C using a feedback-controlled heating system. For surgical preparation, the head was fixed in a stereotactic holder and a trephination was performed over the left hemisphere of the skull, exposing the brain (circular of 3–4 mm diameter between Bregma and Lambda) using a minidrill, and cooling was performed with ACSF during the procedure. The composition of ACSF millimols per litre (mmol/L) was as follows: NaCl 138.4, KCl 3.0, CaCl_2_ 1.3, MgCl_2_ 0.5, NaH_2_PO_4_ 0.5, urea 2.2, and glucose 3.4, pH 7.4, warmed to 37 °C and equilibrated with 5% CO_2_ in O_2_. The dura and arachnoid under the trephination were removed and the exposed cortex was kept moist with ACSF. A barrier of dental acrylic was constructed on the skull around the trephination, providing a 50–100 µL bath for topical application of fluids to a restricted cortical area (Fig. 1B).

### Recording of intracortical direct current potentials, and data processing

An Ag/AgCl reference electrode (containing 2 mol/L KCl) was placed on the nasal bone. For direct current electrocorticogram (DC ECoG) recordings, we used four electrodes (DC1, DC2, DC3, DC4) with tip diameters of approximately 5 µm and resistances < 10 MΩ.

All ECoG electrodes, the CSD elicitation pipette combined with DC1, and a [K^+^]_e_-sensitive electrode combined with the electrode DC3 were located at a depth of 200–250 µm (cortical layer III). DC2 and DC4 were separated from DC1 and DC3 by 400 µm horizontally. The [K^+^]_e_-sensitive electrode contained a Corning 190 K^+^ ion exchanger (WPI 190, Sarasota, FL, USA)^16^ in its tip and was prepared according to previous protocols^9^.

In each mouse, 5–7 CSDs were elicited at 30-min intervals by KCl microinjection (time 0, 30, 60, 90, 120, 150, 180 min) while warmed, carbogen-equilibrated ACSF was maintained in the trephination bath with a continuous flow. If CSDs failed to develop after KCl microinjection at 100 ms, the CSD stimulus was increased in steps of 0.2 s up to 1.0 s of KCl application.

CSDs were evaluated in terms of successful elicitation by KCl application, maximal amplitudes relative to baseline before the steep onset of depolarization, duration at half-maximum amplitude, propagation time from the site of evocation (electrode DC1) to electrode DC3, and threshold of KCl application, if applicable.

To analyze the depression patterns of the ECoG activity associated with spreading depression, DC recordings were resampled offline at a sampling rate of 205 Hz and first detrended by appropriate adaptive filtering, followed by bandpass filtering (0.01–45 Hz). To reveal alternating current (AC)-ECoG activity, the signals were high-pass filtered with a lower frequency limit of 0.5 Hz. This type of analysis also allows the presence of epileptiform electroencephalogram (EEG) to be assessed and seizures to be observed.

### Measurement of the extracellular space volume

The real-time iontophoretic method was used to determine the ECS diffusion parameter volume fraction (α)^17,18^. This parameter indicates the space available for diffusion and is defined as the ratio of the volume of the ECS to the total tissue volume in a representative volume of brain tissue. Briefly, tetramethylammonium (TMA^+^) was delivered into the cortical ECS at a depth of 200 µm using the iontophoretic microelectrode made of theta glass tubing (Science Products GmbH, Hofheim, Germany) filled with 100 mM TMA^+^ (Fluka Chemie AG, Buchs, Switzerland). The concentration of TMA^+^ was measured using a double-barreled TMA^+^-ion sensitive microelectrode^19^ glued to the iontophoretic microelectrode at a distance of 100 µm. The tip of the ion-sensitive barrel was filled with the ion exchanger IE190 (WPI 190, Sarasota, FL, USA), which also senses TMA^+^, and the TMA^+^-sensitive barrel was back-filled with 100 mM TMA^+^ chloride. The reference barrel contained 150 mM NaCl. The TMA^+^ ion sensitive microelectrodes were calibrated in a series of 4 different concentrations of TMA^+^ (0.1, 0.2, 0.4, 0.8 mM) on the background of 150 mM NaCl and 3 mM KCl^20^. Calibration data were fitted with the Nikolsky equation, to determine the electrode slope and interference of saline ions^17^ (Fig. 1C). Prior to tissue measurements, diffusion curves were first recorded in 0.3% agar gel (Sigma Aldrich, Germany) dissolved in 150 mM NaCl, 3 mM KCl, and 1 mM TMA^+^Cl. The microelectrode array was then lowered into the brain cortex to a depth of 200 µm (see Fig. 1D for examples). After the experiments, the diffusion curves were transferred to a PC-based computer and analyzed by fitting the data to the modified diffusion equation using VOLTORO software to obtain the diffusion parameters of ECS in nervous tissue^17^.

### Behavioral analysis in WT and TASTPM animals

The initial effects of sodium thiopental injections on behavior before and during the onset of anesthesia were recorded in some animals of each genotype (WT and TASTPM) and age (3, 6 and 12 months). The effect of sodium thiopental on tail tension, shivering, falling to the side, single jumps and clonic tonic jumps was assessed. Shivering was defined as a constant movement of the head and front paws, similar to a typical response to cold temperatures. Falling to the side was considered when a mouse lost its balance and unexpectedly fell with the whole body to one side, followed by recovery to its initial position. A single jump was defined as a post-injection response in which the animals made a jump lasting at most 1 s, followed by normal behavior. Finally, clonic tonic jumps resembled seizure-like movements manifesting as continuous and uncontrolled jumping throughout the cage lasting from 3 to 30 s. The percentages of occurrence of clonic tonic jumping were calculated.

### Histological preparations and immunohistofluorescence

Male WT mice, (n = 3), male TASTPM mice (n = 3), female WT mice (n = 3) and female TASTPM mice (n = 3) of all ages (3, 6 and 12 months) were perfused using first PBS buffer under deep anesthesia until euthanasia, followed by perfusion with 4% ice-cold phosphate-buffered paraformaldehyde (PFA; Sigma-Aldrich, Saint Louis, MO, USA). Brains were removed, post-fixed in 4% PFA for at least 24 h, equilibrated in 30% sucrose, and frozen at − 80 °C. These mice did not undergo CSD recordings to avoid the interference of the CSD on the histology. Coronal slices of 10 µm thickness were cut using a Leica CM3050S cryostat (Leica Biosystems, Nussloch, Germany). Sections were washed with TBS solution (20 mmol/L Tris base, 137 mmol/L NaCl, pH 7.4) and antigen retrieval was performed with 10 mmol/L citrate buffer (pH 6.0) at 95 °C for 30 min. Sections that were incubated with Galectin-3, CD68, CD39 underwent an additional incubation step with 0.1% NaBH_4_ in distilled H_2_O for 20 min at room temperature to reduce background after being washed with TBS at room temperature. For other antibodies this step was omitted. The sections were then washed with TBS and incubated for blockade with TBS containing 1% Triton X-100 and 10% goat serum (AURION, Wageningen, Netherlands) or 10% donkey serum (Dianova, Hamburg, Germany) for 30 min at room temperature. The sections were then incubated with the primary antibodies overnight at 4 °C. The primary antibodies were rabbit anti-CD39 (1:100, Abcam ab227840), rat anti-CD68 (1:500, Bio-Rad MCA1957GA), rat anti-Galectin-3 (1:500, Invitrogen 14-5301-82), rabbit anti-GFAP (1:100, GeneTex GTX108711), goat anti-Iba1 (1:200, Genetex GTX89792), mouse anti-NeuN (1:100, Millipore MAB377), and mouse anti-P-TAU (Ser202, Thr205) (1:100, Invitrogen MN1020), diluted in TBS containing 1% Triton X-100 and 2% goat serum or 2% donkey serum in a humid chamber. Sections were then washed 3 times with TBS and incubated with the secondary antibody for 2 h at room temperature. Secondary antibodies were raised in the desired species to recognize the primary antibodies and conjugated with Alexa Fluor 488 or 568 (1:200; #A11008, #A11004, #A11001, #A11011, #A11055, #A10042 and #A78946, Thermo Fischer Scientific, San Diego) diluted in TBS containing 1% Triton X-100 and 2% goat serum or 2% donkey serum. The sections were then stained for identification of nuclei with Hoechst 34580 (1:500; Invitrogen, Darmstadt, Germany) diluted in TBS during 10 min at room temperature. Finally, sections were mounted with ProLong Gold (Invitrogen, San Diego, CA, USA) and coverslips, followed by storage in the dark. Negative control experiments were performed without the primary antibodies. Images were captured using a confocal laser scanning microscope TCS SP5 (Leica, Wetzlar, Germany). Contrast and brightness were adjusted using ImageJ^21^.

For labelling of Aβ plaques in combination with immunofluorescence, Congo Red dye (HT60, Sigma Aldrich, Saint Louis, MO, USA) was used prior to the immunolabelling according to the manufacturer’s instructions. In this case, sections were rinsed with tap water for 5 min. The sections were then incubated with Alkaline Sodium Chloride Solution for 20 min, followed by staining with Congo Red Solution for 20 min. They were then rinsed briefly with tap water. Labelling with the appropriate antibodies of interest (Iba1, GFAP or NeuN) was then performed according to the above protocol. Control experiments were performed without the primary antibodies. Images were captured using a confocal laser scanning microscope TCS SP5 (Leica, Wetzlar, Germany). Contrast and brightness were adjusted using ImageJ^21^.

Aβ plaques were quantified by manually counting the number of independent Aβ clusters stained with Congo Red in the entire cortical area (3 independent 10 µm thick sections per mouse, n = 3 mice, see also Supplementary Table 2) of one hemisphere (from the midline to the end of the cortex in the caudal position). A Zeiss microscope (Axioplan 2, objective: Zeiss Plan NEOFLUAR 20x/0.50) was used for plaque counting at 20 × magnification. The entire hemisphere was manually scanned in the same order: from the midline to the end of the cortex, going up and down to evaluate the entire cortical hemisphere.

### Data statistics

Data in line and bar graphs are presented as mean ± standard error of the mean. Scatter plots show the distribution of individual data points. Statistics were performed using: (1) the one-sample t-test against the reference value; (2) Mann–Whitney U test between groups for comparison of means; (3) Fisher’s exact test for comparison of categorical data; (4) one-way-ANOVA for comparison of data distribution; and (5) linear regression to correlate variables. Calculations were performed using InStat (Graph Pad, San Diego, CA, USA). Significance was accepted at a *p*-value < 0.05.

## Results

### Histopathological effects of AD on TASTPM cortex

**Figure 2:**
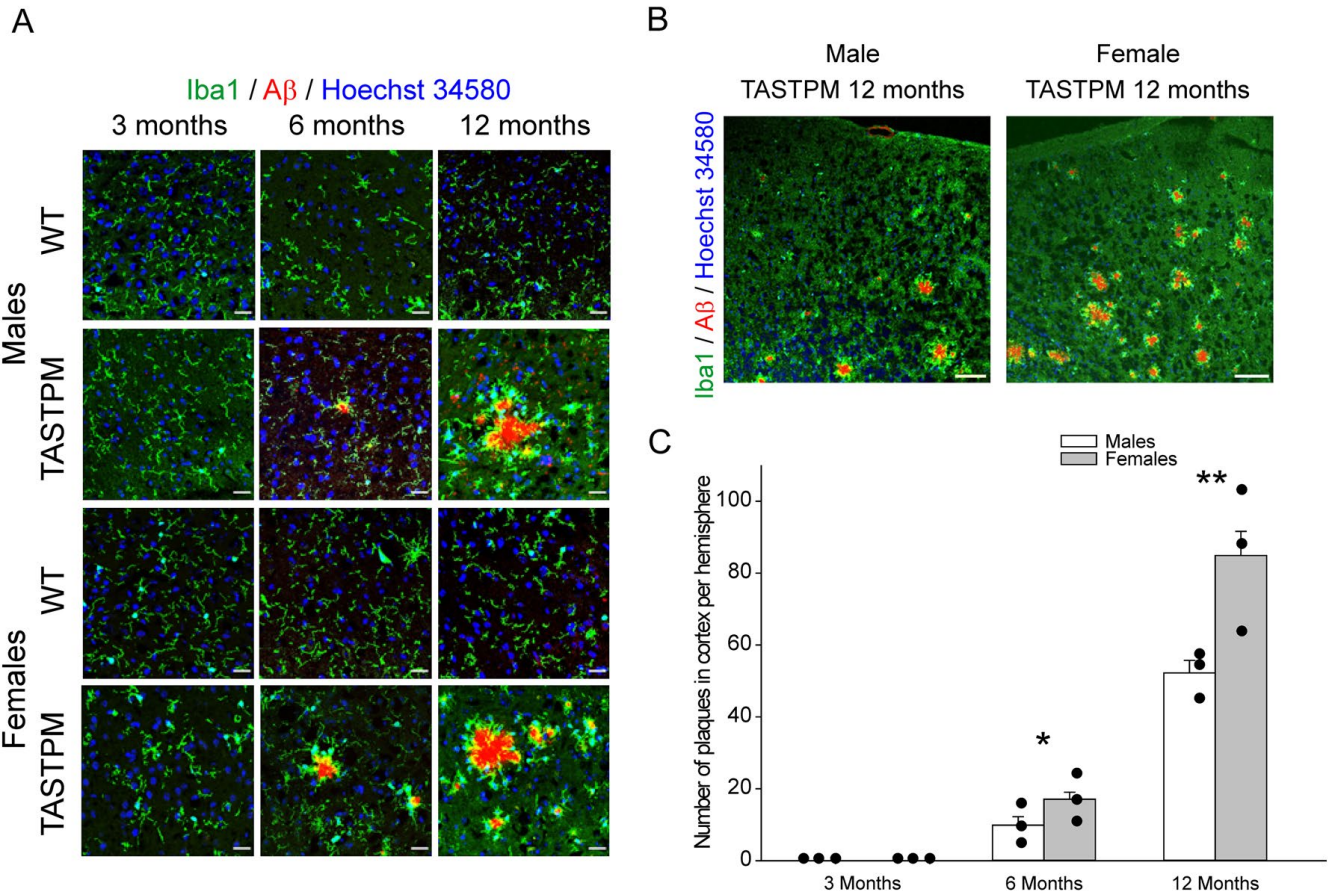
Effect of AD development on mouse cortical histopathology and number of plaques. (**A**) Development of AD with cortical deposits of Aβ. Clusters of microglia around the Aβ in AD (forming a ring) and normal distribution in WT. Microglia was stained by immunolabelling with anti-Iba1 (in green), Aβ with Congo Red (in red) and nuclei with Hoechst 34580 (in blue). Scale bars 25 µm. (**B**) Overview on plaque formation in 12 months old male and female TASTPM mice with a lower magnification. Staining is the same as in A. Scale bars 100 µm. (**C**) Comparison of plaque numbers per hemisphere in male and female TASTPM mice at 3, 6, and 12 months of age. Bars show mean ± standard error (n = 3 mice per group), data dots give the mean values from three independent cortical slices in each mouse. Asterisks denote significant differences between plaque numbers in males and females at the same age (*p* = 0.0315, and *p* = 0.0012, respectively, Mann–Whitney U test).

To investigate the impact of AD on CSD, disease progression in the cortex, characterized by Aβ plaque development and changes in ECS volume, was assessed. Aβ plaques were stained with Congo Red dye and microglial cells were identified by Iba1 immunolabelling (Fig. 2A). A gradual development of AD histopathology in the cortex was observed. At early stages (3 months), TASTPM animals showed no Congo Red signal and microglia showed a homeostatic morphology, similar to the cortex in WT animals at the same age. At intermediate stages (6 months), we found first signs of Aβ plaques in the cortex surrounded by a cluster of microglial cells, but no signs in WT mice. Finally, at advanced stages of AD (12 months), the cortex showed a greater number of plaques surrounded by microglial cells. In contrast, the WT cortices remained unaltered throughout 12 months.

Using Congo Red dye as a marker, we quantified plaque density in one hemisphere per mouse at different stages. Figure 2B shows the occurrence of plaques at 12 months at low magnification, and the difference between sexes in advanced AD is shown. Figure 2C shows the plaque density quantification in all TASTPM mice. Three months: no plaques; 6 months: 9.9 ± 2.4 plaques/hemisphere in males, 17.1 ± 1.9 plaques/hemisphere in females; 12 months: 52.2 ± 3.5 plaques/hemisphere in males and 84.9 ± 6.7 plaques/hemisphere in females. Females had a significantly higher number of plaques than male at 6 and 12 months (*p* = 0.0315, and *p* = 0.0012, respectively, Mann–Whitney U test).

**Figure 3:**
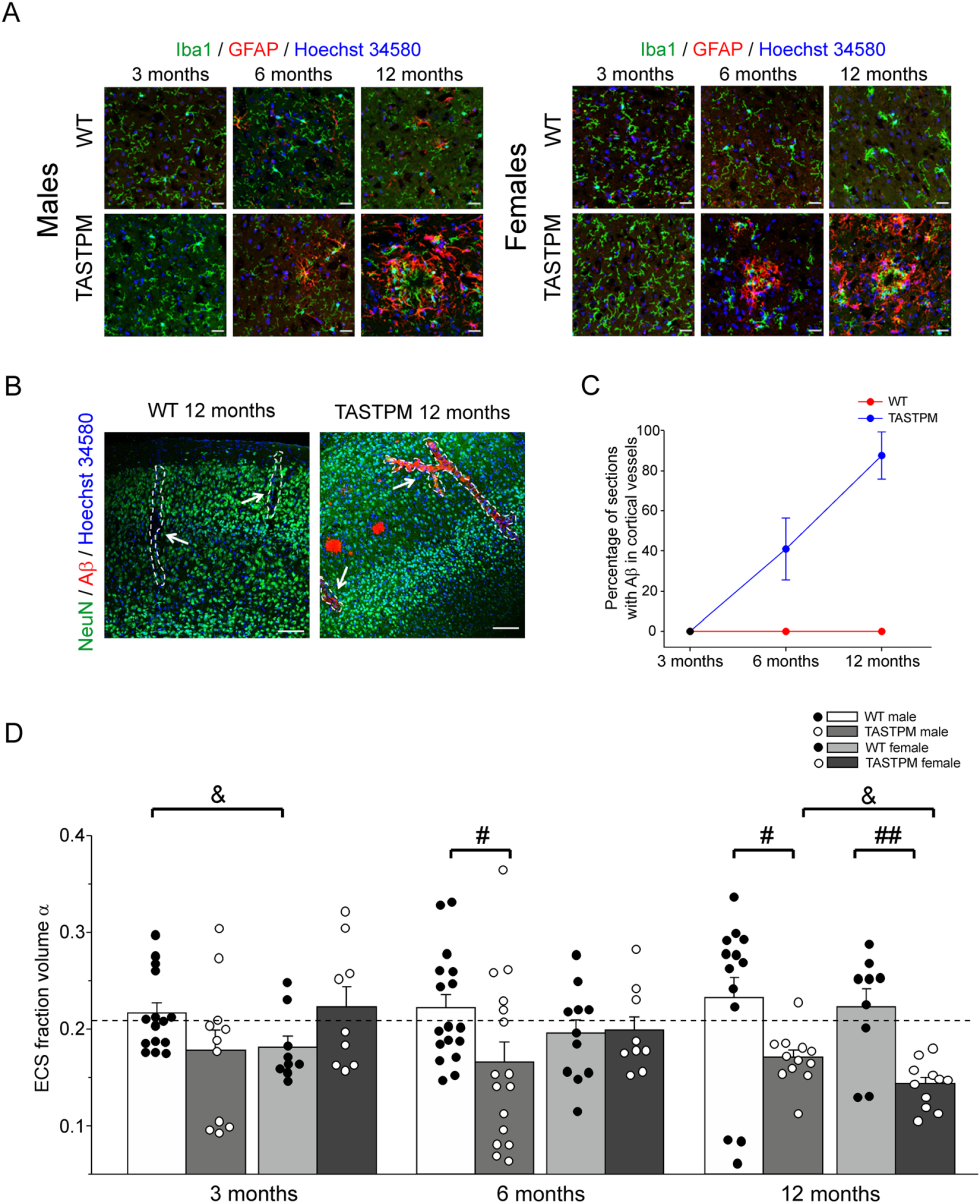
Effect of AD development on cortical astrogliosis, vascular pathology and size of ECS volume fraction α. (**A**) Progressive development of astrogliosis in TASTPM cortex compared to WT. Aβ deposits are primarily surrounded by microglial cells and then by astrocytes. Astrocytes stained with GFAP show the activation of this cell type in AD. Microglia was stained by immunolabelling with anti-Iba1 (in green), astrocytes were stained by immunolabelling with anti-GFAP (in red) and cell nuclei were stained with Hoechst 34580 (in blue). Scale bars 25 µm. (**B**) Examples of Aβ deposits within cortical vessels in TASTPM cortices at 12 months of age. No deposits were found in WT cortical vessels at the same age. Scale bars 100 µm. (**C**) Quantification (percentage) of sections showing positive Aβ labelling in cortical vessels at different ages. The percentage of positive sections is shown as mean ± standard error. (**D**) ECS volume obtained by real-time TMA^+^ iontophoresis in WT and TASTPM mice at ages as in A and B. Bars show mean ± standard error (n = 3–5 mice with 3–4 independent replicate measurements), scatter plots show individual data points. The reference value for ECS in the brain of healthy mice is taken from the literature and set at 0.21 (dashed line). # denotes significant differences between WT and TASTPM at the same age and sex (Mann–Whitney U test), & denotes significant differences between males and females at the same age and genotype (Mann–Whitney U test). If appropriate, Bonferroni correction was performed. One symbol *p* < 0.05, two symbols *p* < 0.01. The one-sample t-test against reference value (0.21) revealed significantly lower ECS volumes in female WT mice at 3 months, and in male and female TASTPM mice at 12 months (*p* < 0.05, and *p* < 0.001, respectively, symbols not shown).

Microglia showed a different distribution according to AD progression (Fig. 2A), as previously described and quantified by other groups for the TASTPM strain, which showed a statistically significant increase in cortical microglia^22^. Another notable characteristic was progressive astrogliosis. In agreement with a previous study^23^, we confirmed that healthy WT cortices do not express GFAP, but during AD progression there was a gradual activation of astrocytes expressing GFAP in the vicinity of Aβ plaques (Fig. 3A), similar to previous findings^22^. In addition, we observed Congo Red signal in large blood vessels of TASTPM cortices (Fig. 3B), with stronger deposition of Aβ in older animals with advanced AD pathology (Fig. 3C). We also assessed Tau pathology, another hallmark of AD. Confirming previous observations^24^, we detected P-TAU-positive cells in advanced stages of AD (Supplementary Fig. 1). As the TASTPM model does not have direct genetic alterations for TAU protein, Aβ plaques may have triggered Tau pathology.

The sum of all morphological changes may have an impact on the volume of the ECS. To determine changes in ECS, we used the TMA^+^-sensitive electrode method, which estimates the ECS fraction by the diffusion parameter α (volume fraction). In WT mice at all ages, the measured mean value of α was close to the literature reference^18^ (dashed line) although at 3 months female WT mice had a significantly lower α than male WT mice (see symbol & in Fig. 3D). The one-sample t-test revealed significantly lower ECS volumes compared to literature^18^ in female WT mice at 3 months, and in male and female TASTPM mice at 12 months (*p* < 0.05, and *p* < 0.001, respectively). In male mice (first two columns at 3, 6, 12 months) α was smaller in TASTPM mice than in WT mice, with a trend at 3 months and statistical significance (#) at 6 and 12 months. In female mice, α was significantly lower in TASTPM mice than in WT mice only at 12 months. Thus, the progressive decrease in α suggests progressive shrinking of ESC volume with the development of AD pathology (Fig. 3D), with an earlier effect in male TASTPM mice. These changes may affect the dilution of ions and make them more concentrated favoring changes in cortical excitability.

**Figure 4:**
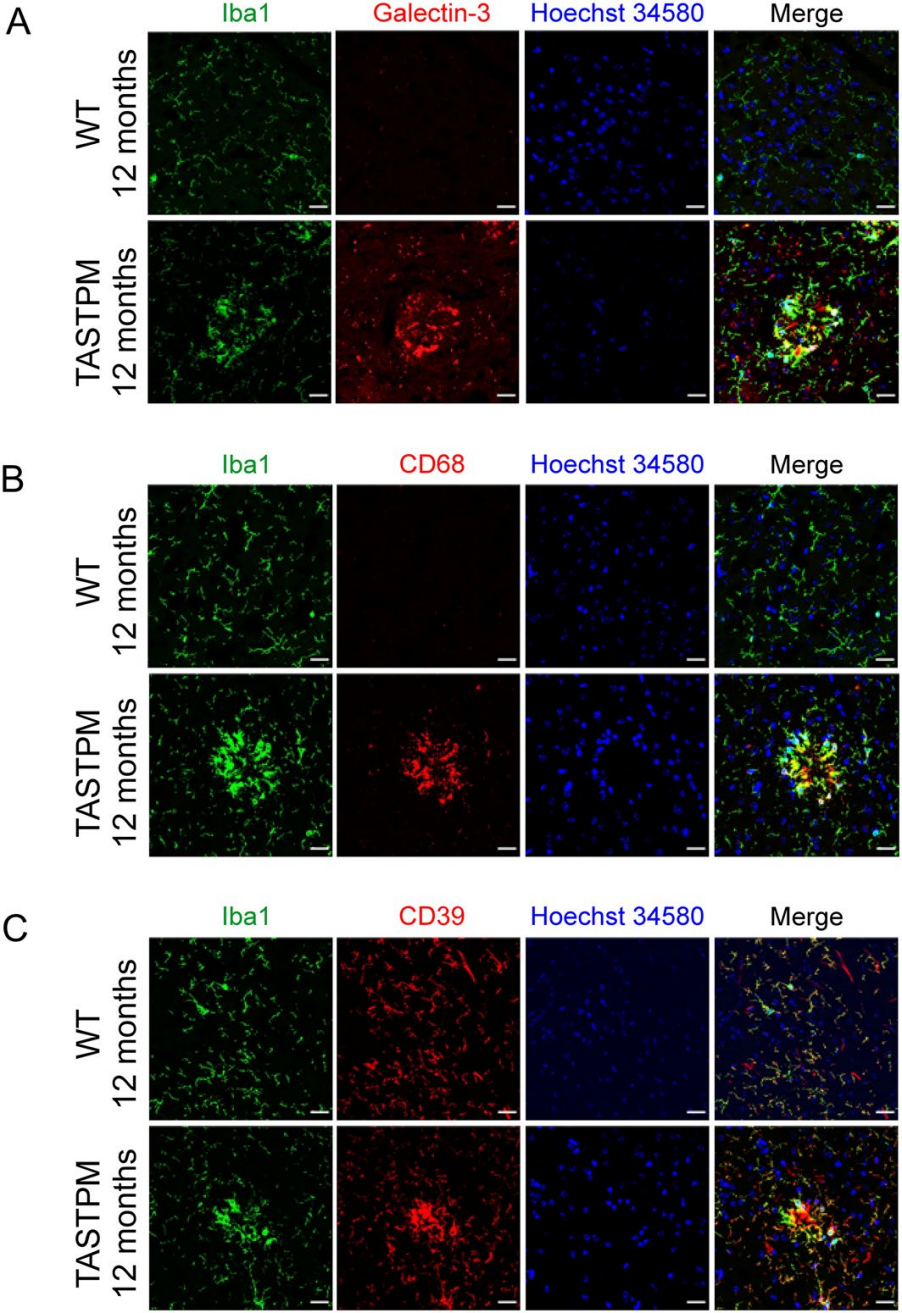
Functional characterization of microglia in TASTPM and WT animals at 12 months of age. Microglia localized by immunolabelling with anti-Iba1 (in green). Functional characteristics such as chemotaxis (expression of Galectin-3) (**A**), macrophagia (expression of CD68) (**B**) and increased metabolic activity (increased intensity of CD39) (**C**) were identified by immunolabelling with anti-Galectin-3, anti-CD68 and anti-CD39 respectively (in red). Nuclei were stained with Hoechst 34580 (blue). Scale bars 25 µm.

Microglia can be active or in a resting phenotype. To determine the role and status of microglia, different markers for specific activity characteristics were immunolabelled in the cortices at 3, 6 and 12 months (Fig. 4). Due to the incompatibility of the secondary antibody Alexa 568 (red color) and the Congo Red dye (also red), the Congo Red positive plaques could not be directly visualized in these sections. However, as shown in Fig. 2, Iba1-labeled microglial clusters developed only around plaques. Therefore, the round clusters in Fig. 4 are considered to be plaque-associated clusters. Figure 4 shows cortical sections of WT and TASTPM at 12 months. Only microglial cell clusters around AD plaques expressed Galectin-3, a molecule involved in the chemoattraction of monocytes and macrophages (Fig. 4A). In addition, clustered microglial cells expressed CD68, a typical lysosomal marker that manifests the macrophage behavior of microglia (Fig. 4B). Finally, the marker CD39, expressed by microglia but at rest restricted to the ramifications, showed an expression also in the cell body of those microglial cells forming clusters (Fig. 4C). At 3 months AD, microglia remained homeostatic (similar to WT shown in Fig. 4) and at 6 months AD, a similar picture to 12 months was seen only in the small clusters under development (data not shown). Together these characterizations showed the transition of resting microglial cells to a pro-inflammatory phenotype in the cortex of TASTPM mice, thus indicating the development of neuroinflammation under AD conditions.

### Heterogeneous development of CSDs in WT and AD

To investigate the impact of the histopathological changes on cortical homeostasis and excitability, CSDs were recorded in vivo in mice anaesthetized with sodium thiopental. The use of sodium thiopental allowed the recording of CSDs because it maintains NMDA receptor-mediated neurotransmission, a prerequisite for CSDs. However, sodium thiopental evoked some behavioral traits, which will be described and analyzed in the next section. In WT and TASTPM animals, males and females, at different ages (3, 6 and 12 months), CSDs were elicited to elucidate a possible correlation between the AD development and progression with CSD elicitation and parameters. Table 1 summarizes the data obtained for the CSD parameters (amplitude, propagation velocity and threshold as time of KCl application for CSD elicitation, success rate in CSD elicitation) according to genotype (WT and TASTPM), age (3, 6 and 12 months) and sex (male and female).

**Table 1:** CSD parameters in WT and TASTPM animals, male and female, at 3, 6 and 12 months of age. Amplitudes, propagation velocities and KCl application time are expressed as mean ± standard error.

Sex	Males						Females					
Age	3 months		6 months		12 months		3 months		6 months		12 months	
Genotype	WT	TASTPM	WT	TASTPM	WT	TASTPM	WT	TASTPM	WT	TASTPM	WT	TASTPM
Percentage of CSD out of total attempts (%)	41	73	80	66	38	33	89	71	45	50	71	63
CSD amplitude (mV)	10.03 ± 2.29	11.38 ± 2.75	9.13 ± 1.39	8.19 ± 1.58	12.53 ± 2.82	11.62 ± 4.81	14.22 ± 0.7	9.65 ± 2.66	13.76 ± 0.81	10.06 ± 2.84	7.93 ± 3.09	9.04 ± 3.54
CSD propagation velocity (mm/min)	1.98 ± 0.28	1.87 ± 0.34	1.95 ± 0.19	2.56 ± 0.22	1.44 ± 0.35	2.19 ± 0.63	2.34 ± 0.28	2.18 ± 0.3	1.9 ± 0.16	2.81 ± 0.66	2.28 ± 0.38	2.47 ± 0.45
KCl threshold (ms)	390 ± 126.89	300 ± 65.47	360 ± 166.13	520 ± 128.06	237.5 ± 102.82	391.67 ± 75.74	220 ± 80	557.14 ± 119.24	400 ± 300	266.67 ± 80.28	308.33 ± 154.07	412.5 ± 95.31

**Figure 5:**
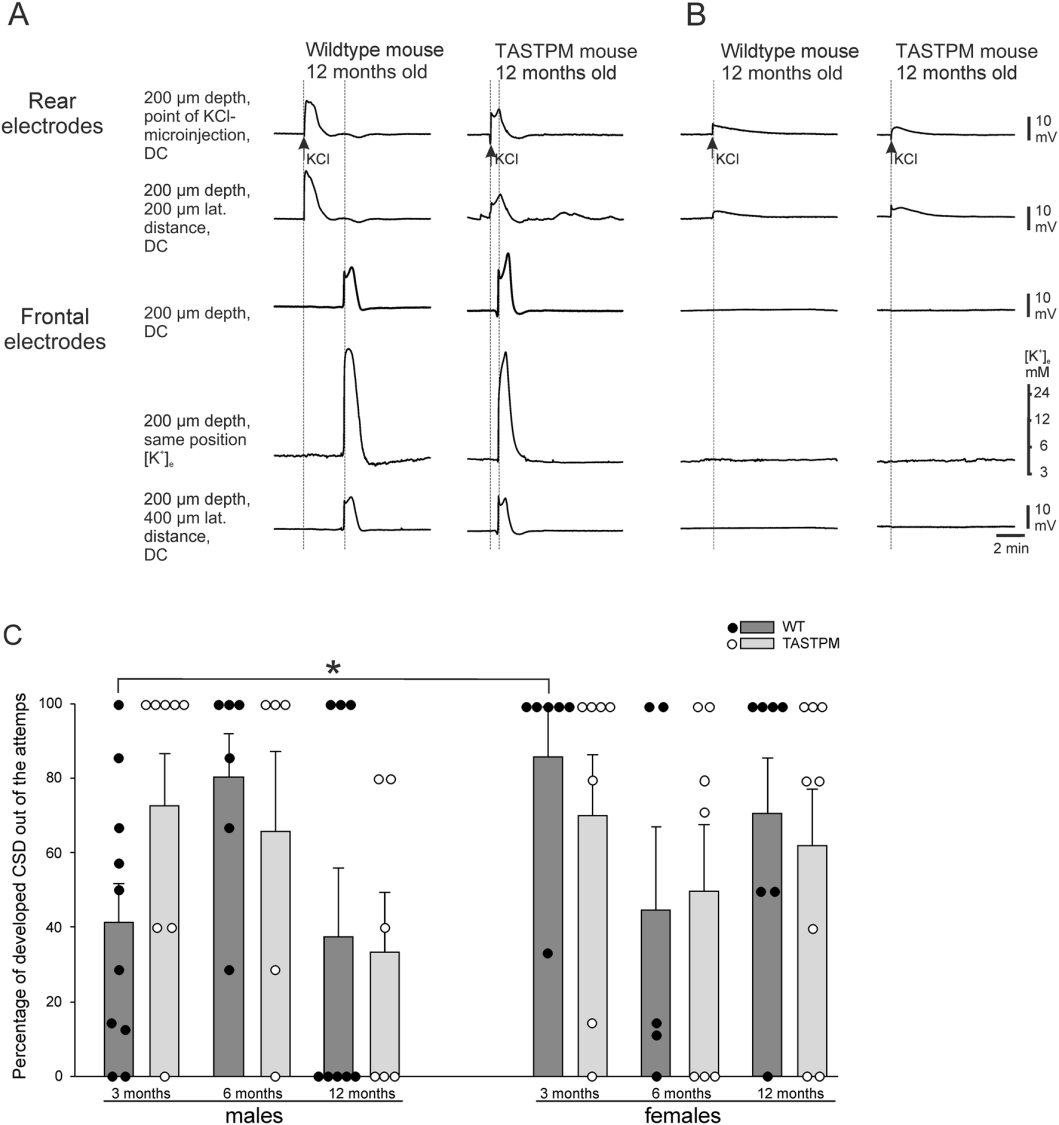
Attempts to induce CSD by KCl failed in some cases in both WT and TASTPM mice. (**A**) Example of a propagating CSD after KCl injection (arrow) in 12-month-old WT and TASTPM animals. (**B**) Example of an attempt to induce a CSD resulting in a local depolarization in this area without a propagating wave. Similar results to A and B were observed in 3-, 6- and 12-month-old males and females. In A and B, DC recordings are shown, the beginning and the maximum of the CSD-related DC shifts are accentuated by thin dotted lines, and a [K^+^]_e_ sensing electrode is also placed to record the CSD-related increase in [K^+^]_e_. (**C**) Percentage of real propagating CSDs from ignition trials in WT and TASTPM males and females at different ages. If no CSD could be elicited, the data dots are at the 0-line. Bars represent mean ± standard error, dots represent individual data points. Statistical significant differences were seen only between 3 months WT males versus females (*p = 0.0103, Mann–Whitney U test).

Surprisingly, mice did not consistently show propagating CSDs after K^+^ microinjection, in contrast to rats. In both WT and TASTPM mice only part of the animals showed typical CSD waves propagating from the rear to the frontal electrode with an amplitude greater than 5 mV (parameters adopted from Somjen^25^) regardless of sex and age (Fig. 5A). In another proportion of animals of all groups K^+^ microinjection induced only a local depolarization in the elicitation area (posterior), but not a spreading wave along the cortex to the frontal area, even when KCl was applied longer (Fig. 5B).

The percentages of successful attempts per animal to elicit propagating CSDs are shown in Fig. 5C for males and females. Each dot represents one animal. Some animals showed always propagating CSDs after each K^+^ injection (see dots at 100%). In other animals only some of the K^+^ injections induced propagating CSDs (dots between 0 and 100%). Some animals showed no propagating CSD after KCl (dots at 0%), even after injection of KCl for 1.0 s. This stimulus was considered sufficient because 90% of all manifest CSDs were elicited by KCl injections < 500 ms. Only at 3 months of age female WT mice showed significantly more attempts with positive outcome than male WT mice (*p* = 0.0103, Mann–Whitney U test). Other comparisons between WT and TASTPM mice, males and females, and between different ages showed no significant differences (ANOVA and Mann–Whitney U test).

In the animals, which showed full-blown propagating CSDs, no significant differences were found for all of the parameters shown in Table 1. Thus, susceptibility to CSD was similar in TASTPM and WT at all ages and in both sexes. Therefore, although there is a large impact of AD neurodegeneration in the cortex, the damage did not affect the development of CSD compared to age-related WT.

### Sodium thiopental induced abnormal EEG activity but manifested hyperexcitation in TASTPM animals

The rate of unsuccessful CSD elicitation by KCl (Fig. 5C) raised the question, of whether another type of cortical activity could interfere with the CSD outcome. In literature, a link between AD and epilepsy is described^26^. However, the coexistence of epilepsy and CSD is under debate^9,11^. We found that the use of sodium thiopental induced specific hyperexcitation during the beginning phase of anesthesia. Shortly after the injection of sodium thiopental many WT and TASTPM mice showed transient hyperexcitable behavior, characterized by tail tensing, shivering, falling to side, single jumps and clonic jumps, usually lasting several minutes before anesthesia was deep and animals were unconscious and immobile.

**Figure 6:**
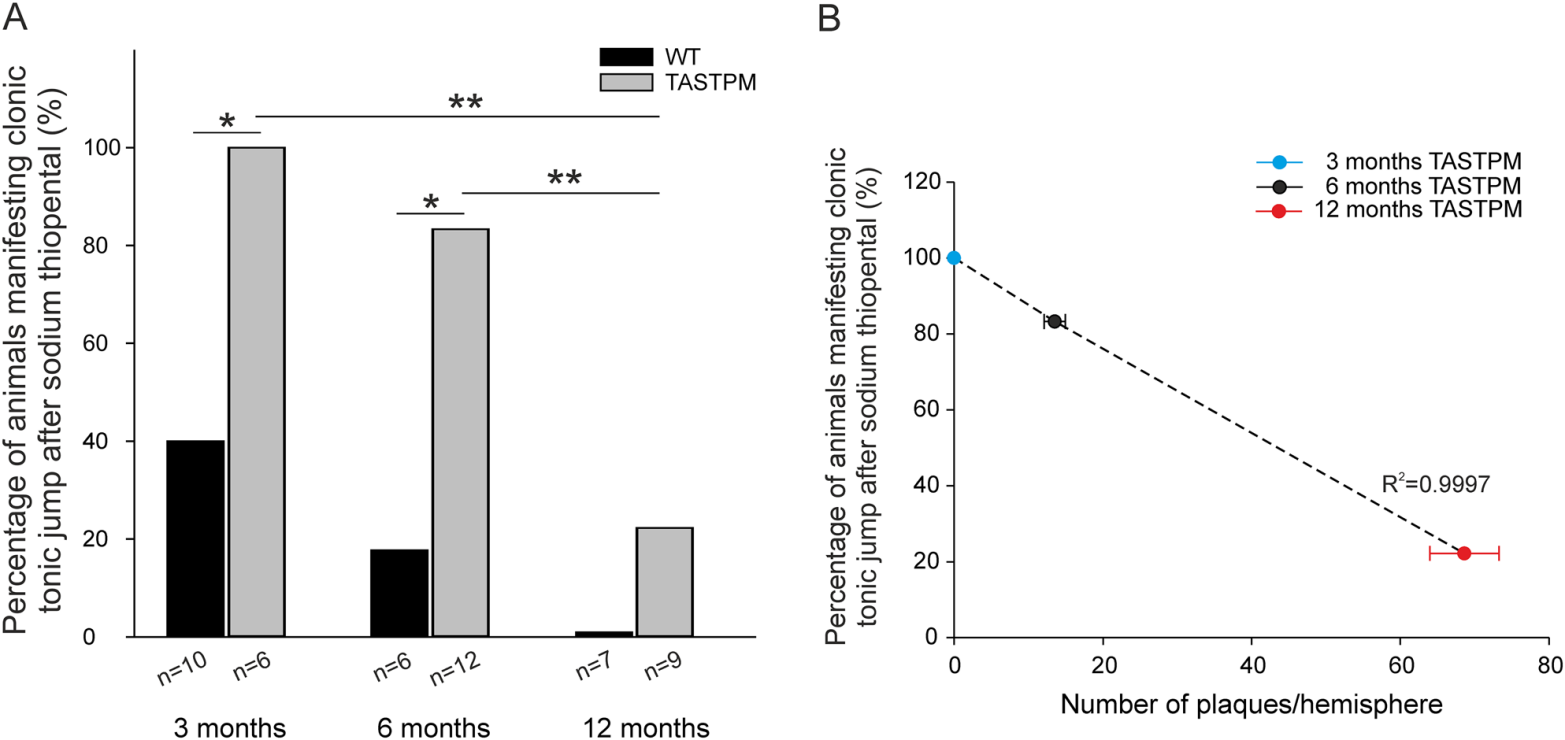
The use of sodium thiopental induced clonic-tonic jumping during the excitation phase of anesthesia in a minority of WT and in TASTPM mice. (**A**) Clonic-tonic jumping occurred significantly more often in TASTPM compared to WT mice at the ages of 3 and 6 months (*p* = 0.0308, *p* = 0.0128, respectively, Fisher’s exact test). With aging the proportion of TASTPM mice that show this trait significantly decreases (3 vs. 12 months *p* = 0.0048; 6 vs. 12 months *p* = 0.0092, Fischer’s exact test). (**B**) The correlation of the percentage of clonic-tonic jumping during anesthesia at different ages and the mean number of Aβ plaques in TASTPM mice shows a linear regression. No plaques were found in WT mice at any ages, therefore these data are not shown. Male and female mice are pooled, data points represent mean values ± standard error.

The most remarkable effect of sodium thiopental was the appearance of transient clonic-tonic jumping. At the age of 3 months a minority of the WT mice but all of the TASTPM mice showed transient clonic-tonic jumping (Fisher’s exact test, *p* = 0.0308) (Fig. 6A). At 6 months less WT and TASTPM mice showed transient clonic-tonic jumping (Fisher’s exact test, *p* = 0.0128), and at 12 months only a few TASTPM mice showed this behavior. The statistically significant difference between WT and TASTPM mice at the earlier stages associates the clonic-tonic behavior with AD pathology at early stages. In order to correlate the clonic-tonic behavior with the Aβ deposition, a linear regression has been performed and we observed a higher clonic-tonic behavior when no plaque has been detected in cortex and Aβ should still be soluble (Fig. 6B).

**Figure 7:**
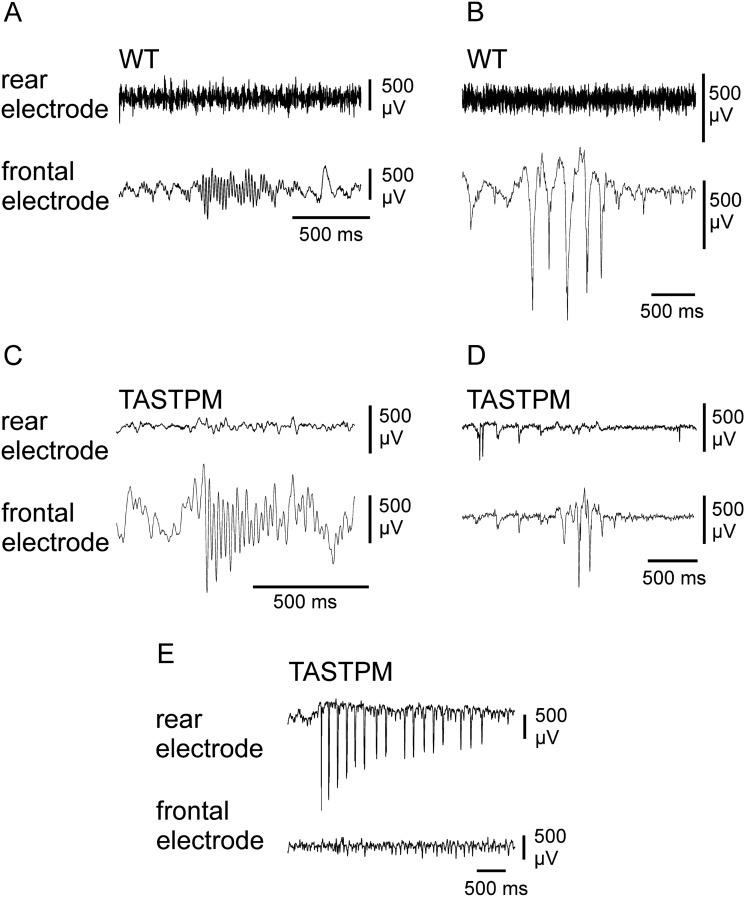
The use of sodium thiopental as an anesthetic produced abnormal EEG patterns in both WT and TASTPM animals. EEG evaluation revealed three main patterns of pathophysiological activity: sharp waves, spindle activity and seizures. Seizure activity was only observed in TASTPM mice. (**A**) Example of spindle activity in WT animals. (**B**) Example of sharp wave activity in WT animals. (**C**) Example of spindle activity in TASTPM animals. (**D**) Example of sharp wave activity in TASTPM animals. (**E**) Example of seizure in TASTPM animals. All patterns shown correspond to 12-month-old animals, but these patterns were observed at all ages.

During recordings in anaesthetized mice, we found in the continuous EEG recordings short episodes of abnormal patterns with the presence of spindles and sharp waves in all animals (100%), WT and TASTPM, irrespective of sex and age (Fig. 7A–D). However, the background of ictal activity seen in the clonic-tonic jumps was also manifested in some TASTPM animals as discrete seizures that could only be recorded in this group (27% at 3 months, 33% at 6 months, 14% at 12 months) but not in WT mice (Fig. 7E). Taken together the behavior and the EEG recordings showed evidence for hyperexcitability in TASTPM mice.

## Discussion

**Figure 8:**
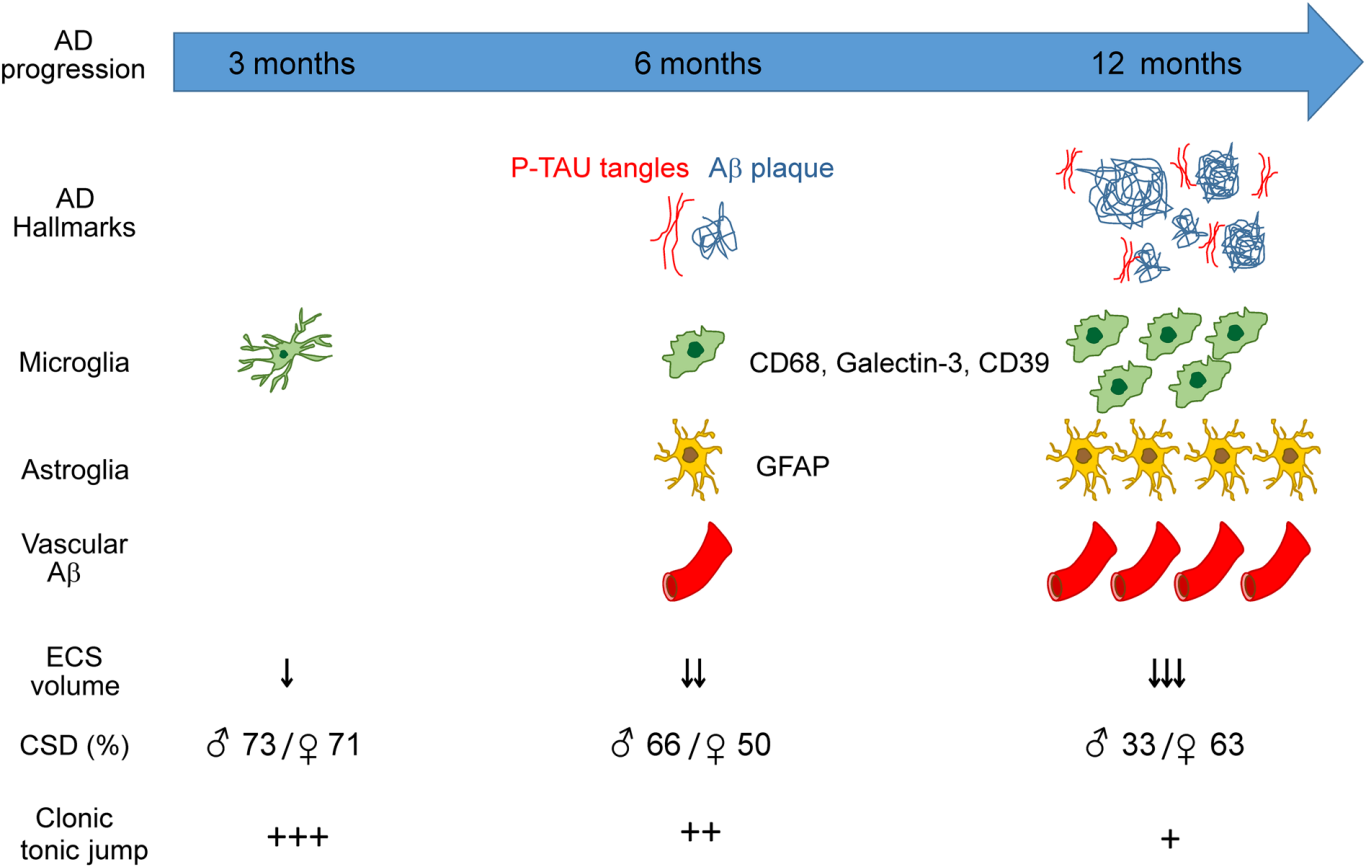
Summary of the data showing the development of AD and its histopathological traits and electrophysiological features (CSD and ECS) and the clonic tonic jumping underneath sodium thiopental anesthesia at ages of 3, 6, and 12 months after birth of TASTPM mice.

This study further characterized the TASTPM model histologically and evaluated whether histopathological changes correlate with electrophysiological data. All histopathological findings showed significant cellular changes during AD progression accompanied by neuroinflammatory features. In addition, we showed shrinkage of ECS volume, suggesting changes in the extracellular space. However, measuring CSD elicitation capability as a functional parameter for changes in homeostasis did not reveal a correlation between histological manifestation of AD pathology and CSD alterations. In addition to genotype, sex and age must be taken into account. While the induction of abnormal EEG effects by sodium thiopental precluded the in-depth analysis of epileptic discharges in the anesthetized animals, TASTPM mice showed epileptic behavioral changes after initiation of the anesthesia protocol, indicating hyperexcitability and risk of seizures in TASTPM mice (see also Fig. 8).

As described previously^13,14^, TASTPM mice developed Aβ deposits with time, similar as other AD models^27^. Consistent with a previous study on TASTPM mice^28^, the first plaques were detected at 6 months of age, and much larger plaques were observed at 12 months of age. Possibly, the mutation leads to an overproduction of Aβ peptide that is initially kept in a soluble state^13^. WT mice did not show Aβ labelling at any age. Female mice showed more plaques than male mice agreeing with previous findings^14^, in line with observations that female patients are more severely affected by AD than male patients^29,30^. Presumably, differences in hormone levels influence disease progression. Estrogens protect female mitochondria from Aβ toxicity but this protection is lost in old AD patients^30^.

For the first time, we detected Aβ labelling in the brain vessels of 6- and 12-month-old TASTPM mice. Since vascular accumulation of Aβ was also found in APP transgenic mice^31^, vascular impairment may contribute to AD. We also found P-TAU tangles in TASTPM animals at 6 and 12 months of age confirming previous reports that some neurites of TASTPM mice at 6 and 8 months of age are P-TAU-positive^24^. However, P-TAU-positive cells were not localized in the close vicinity of plaques (see also Supplementary Fig. 1). As the TASTPM model has only mutations for the Aβ cascade, Tau pathology may have been triggered by the toxic Aβ deposition in the cortex^32–36^. Thus, TASTPM is a suitable preclinical AD model, showing initially amyloidosis without Tau pathology and both Aβ plaques and P-TAU tangles at advanced ages^37^.

In addition to Aβ plaques and P-TAU tangles, the hallmarks of AD, we confirmed the presence of neuroinflammation consisting of microgliosis along with activation of microglia, and astrogliosis. Glial cells surrounded Aβ plaques. The role of glial cells remains partly unclear^38–42^. They may form a putative glial scar that separates areas with Aβ deposits from healthy areas and/or combats the toxicity induced by AD, and microglia may attack the plaques. However, activated glial cells may also have negative effects and contribute to AD progression^4^.

Positive staining for the markers Galectin-3, CD68 and CD39^43^ showed polarization of microglia into an activated conformation. Galectin-3 is involved in the chemoattraction of monocytes and macrophages^44,45^. In TASTPM mice, we describe for the first time an AD-induced Galectin-3 expression in some of clustered microglial cells surrounding Aβ plaques. Most likely Galectin-3 recruits more microglia to the plaques. CD68 is a classical marker of the macrophage status of microglia^46^. Similar to a previous study^47^ we detected CD68 in clustered microglia around the plaques. However, it cannot be excluded that some of the CD68-positive cells were infiltrating macrophages from the periphery^48^. We found that microglial cells without plaque contact only expressed Iba1, but not CD68. Therefore we assume that CD68-positive microglial cells attempt to combat the plaque by phagocytosis^49,50^. However, the phagocytic status may not be strong enough for clearance, and Aβ deposition is achieved. Possibly, microglial cells become senescent and the rate of phagocytosis may be lower as AD progresses^49,51,52^. CD39 is an ATPase involved in ATP hydrolysis and energy metabolism, and is required for microglial function^53^. During activation of microglial cells CD39 hydrolyzes more ATP^53^. While CD39 upregulation has been described in another AD model^54^, we studied this marker for the first time in TASTPM mice. In WT mice, microglia showed co-localization of CD39 and Iba1 in the ramifications. However, in TASTPM mice, large and bright CD39 expression was also found in the cell body, suggesting that activated clusters of microglial cells at the plaques required increased energy consumption to maintain a high metabolic rate^55,56^. CD39 has also been proposed to be involved in ATP-induced chemotaxis^57^, which may indicate cooperation with Galectin-3. Taken together, these markers show a transition from homeostatic microglia to a neuroinflammatory microglia signature of AD and aging^43^.

AD pathology in TASTPM animals also activated astroglia. Under normal circumstances mouse cortical astrocytes do not express the GFAP marker but they start to express GFAP in pathological or aging conditions^23^. We did not identify GFAP-positive cells in the WT cortex, but the cortex of TASTPM mice showed small clusters of GFAP-positive cells around developing plaques in 6-month-old mice and extensive GFAP-positive astrogliosis in 12-month-old cortices. The data suggest a reactive activation of astrocytes throughout the cortex, especially surrounding plaques beyond the microglial ring (see Fig. 3A). The exact role of astrocytes in AD is still partly unclear^58–60^. Astrocytes are involved in many physiological functions including blood brain barrier formation, neurotransmitter clearance, uptake of ions from the synaptic cleft, and water homeostasis^61,62^. As part of the glial scar, astrogliosis may be protective by isolating healthy areas of the cortex from Aβ pathology, but other authors have postulated that astrocytes may be involved in Aβ production^63,64^.

The histopathological changes (Aβ plaque formation, microglial activation and astrogliosis) may not only alter the cellular components but also change the ECS. Using the TMA^+^ diffusion method, we found a reduction in the ECS fraction in TASTPM mice consistent with the only other report in another AD model^65^. Although the age of the animals and the mouse strain were different in both studies, we found similar values of α. Notably, at least in male mice, the shrinkage of the ECS volume occurred before the histological changes became manifest. Thus, abnormalities in ECS volume may precede plaque formation.

In order to combine the histopathological effects of AD and changes in the ECS with a functional assessment of cortical homeostasis, we recorded CSD waves. To compare young adult, mature adult and late adult mice^66^, we used age groups of 3, 6 and 12 months old mice. Female mice are mature at 3–4 months, sexually mature by 6 months, premenopausal at around 9 months, in the menopause at the age of 12 months, following a reproductive senescence transition between 9 and 12 months^67^. We assume that putative effects of hormonal transitions affect AD pathology (see above) and the development of CSD since publications have shown the influence and increase in excitability and rate of CSD induced by female hormones^68^, fitting to our observations of CSD development and propagation after KCl microinjection. Interestingly, the more pronounced AD pathology in female mice was not mirrored by any major changes in CSD parameters.

Interestingly, CSD susceptibility showed remarkable variability in our experiments. Within almost all experimental groups we found animals in which CSDs were either elicited at all attempts, or only in some attempts, or not at all. Due to this considerable variability, the only significant difference was that at 3 months of age female WT mice showed significantly more attempts with positive outcome than male WT mice. Other comparisons between WT and TASTPM mice, males and females, and between different ages showed no significant differences. Thus, susceptibility to CSD was similarly variable in TASTPM and WT at all ages and in both sexes. These data exclude the overall conclusion that AD influenced the likelihood for CSD elicitation by cortical K^+^ injection, in agreement with a previous study on CSD recording in another AD model where no differences were seen due to AD development and age^69^.

It is possible that the relative weight of individual factors (age, AD, sex) determines the CSD susceptibility in individual animals. In rats, aging reduced the susceptibility to CSD^70–72^. This may be due to augmented production of reactive oxygen species, suppression of the expression of P/Q voltage gated calcium channels or an impairment of the glutamate signaling^72^. An analogous effect in mice is likely, since we have seen local, non-propagating depolarizations as described by Hertelendy et al.^72^ both in aged WT and in TASTPM mice (see also Fig. 5B). On the other side may be factors which increase neuronal excitability, such as the early presence of soluble Aβ in TASTPM mice with subsequently increased neuronal excitability^26,73–75^. Other possible reasons for hyperexcitability are a loss of inhibitory interneuron function and/or the release of excitatory cytokines or neuropeptides such as IL-1β, substance P, or CGRP from reactive glial cells or from other cells, rendering neurons hyperexcitable in AD^8,9,76^. Notably, however, we did not see reactive glial cells in the youngest mice^10^.

A further possible reason for a reduced CSD elicitation rate could be the presence of another type of pathological activity in the cortex such as epileptic discharges. In fact, Busche and colleagues^26^ found that the application of soluble Aβ into hippocampus in vivo induced hyperexcitability that mimics ictal activity mediated by Ca^2+^ currents^26^. For decades, the coexistence between CSD and epilepsy has been matter of debate. Bures et al. presumed that the presence of epilepsy would interfere with the development of CSD, i.e. CSD should not be possible in epileptic foci^11^. Thus, following Bures et al.^11^, failure of propagating CSDs may result from epileptic discharges. However, the dogma of Bures et al. was questioned by our previous study in which we observed a coexistence of ictal discharges and CSD after application of CGRP to the cortex^9^. Still the dogma of Bures could explain why CSDs were not always inducible in TASTPM cortices.

In line with this, we expected the development of epileptic discharges induced by AD. Unexpectedly, however, we found that the use of sodium thiopental also induced aberrant EEG with the development of sharp waves and spindles in both healthy WT and in TASTPM animals. Therefore, an in-depth evaluation of excitability patterns in WT and TASTPM mice was impossible, although discrete seizures were detected in TASTPM animals at 3, 6 and 12 months of age (see also Fig. 7E). However, this sodium thiopental effect has been previously described in animals^77^ and patients^78,79^, showing that drug-induced EEG patterns that can be confused with ictal activity or epilepsy.

However, observation of behavior after sodium thiopental anesthesia revealed consistent differences between TASTPM and WT mice. As reported previously^80^, sodium thiopental can induce a transient hypoxia, characterized by hyperexcitability and epileptic discharges (consistent with TASTPM behavior), before the deep narcotic effect, with blockade of motor activity (consistent with the behavior of WT mice). These observations clearly indicate hyperexcitability in AD brains. Since manifest epileptic activity was never observed during daily cage inspections in the animal facility, it is possible that such hyperexcitability does not trigger manifest seizures in the absence of an additional stimulus, and that this behavioral difference was detected only in the transient excitatory phase of sodium thiopental (indeed, an initial transient excitatory phase is known for many anesthetics, but the reasons are unknown).

## Conclusions

This work adds novel data to the AD phenotype of the TASTPM model, e.g. the more detailed characterization of microglial and astroglial markers and of the ECS volume. This may identify targets for intervention. Our work also highlights that the study of the functional phenotype must take into account maturation, aging and sex differences. It also shows that dramatic changes in cortical histopathology not necessarily correlate with the changes in electrophysiology, namely shape and rate of elicitation of CSD. Nevertheless, our study provides evidence for the early appearance of pathological changes such as ECS volume shrinkage and hyperexcitability, suggesting that these changes may not only be the consequence of AD pathology, but may also be involved in the pathogenesis of the disease. Therefore, it should be investigated whether non-invasive measurements of these parameters could be useful in human studies to investigate the pathogenic mechanisms of AD development.

### Supplementary Information

Supplementary Figure 1.

Supplementary Table 1.

Supplementary Table 2.

## Data Availability

The datasets generated during and/or analyzed during the current study are available from the corresponding author on reasonable request.
