# Supplementary material for: Neuropathological changes in the TASTPM mouse model of Alzheimer’s disease and their relation to hyperexcitability and cortical spreading depolarization
Source: Sci Rep. 2024 Mar 27;14:7224. doi: 10.1038/s41598-024-57868-4 (PMC10973448; doi:10.1038/s41598-024-57868-4)
Supplement: Supplementary file 1 — Supplementary Figure 1. [file 41598_2024_57868_MOESM1_ESM.docx]

Supplementary Figure 1


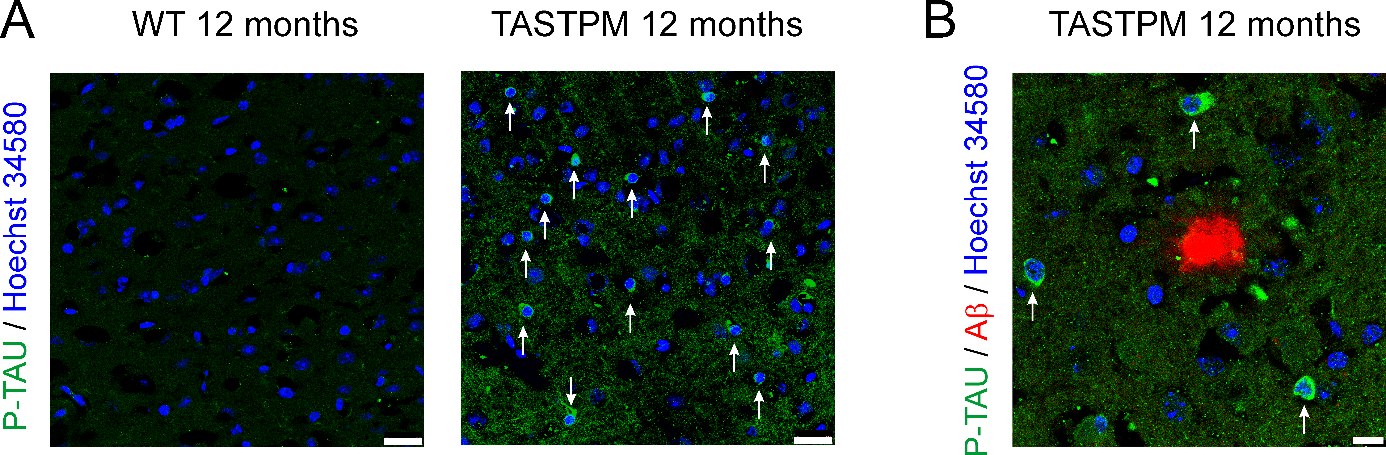


Supplementary Figure 1: Development of P-TAU tangles in TASTPM animals. (A) Presence of P-TAU-positive cells in 12-month-old TASTPM cortices. No signal of P-TAU was obtained in WT 12 month old cortices. Similar results were obtained in 6-month-old animals. Arrows indicate P-TAU-positive cells. Anti-P-TAU signal observed in green and nuclei stained with Hoechst 34580 (in blue). Scale bars 25 µm. (B) Example of P-TAU-positive cells in the vicinity of a plaque. Similar results were obtained in 6-month-old TASTPM animals. Arrows indicate P-TAU-positive cells. P-TAU identified by immunolabelling with anti-P-TAU (in green), Aβ plaques stained with Congo Red (in red) and cell nuclei stained with Hoechst 34580 (in blue). Scale bar 10 µm.
