# Supplementary material for: Neuropathological changes in the TASTPM mouse model of Alzheimer’s disease and their relation to hyperexcitability and cortical spreading depolarization
Source: Sci Rep. 2024 Mar 27;14:7224. doi: 10.1038/s41598-024-57868-4 (PMC10973448; doi:10.1038/s41598-024-57868-4)
Supplement: Supplementary file 2 — Supplementary Table 1. [file 41598_2024_57868_MOESM2_ESM.docx]

Supplementary Table S1

Overview on the numbers of mice used in each animal group. Mice were used for measurement of ECS volume and for CSD recordings. Lower numbers in the columns Volume measurement and Electrophysiology are due to accidental failure of TMA^+^ electrodes.

| **Age** | **Group** | **No. of mice** | **Anesthesia** | **Observation during anesthesia** | | **Volume measurement** | **Electrophysiology**  **CSD + ECoG** |
| --- | --- | --- | --- | --- | --- | --- | --- |
|  |  |  |  | **Visual** | **Video** |  |  |
| **3 months** | WT males | 10 | 10 | 10 | 10 | 5 | 10 |
|  | WT females | 6 | 6 | 6 |  | 3 | 6 |
|  | TASTPM males | 8 | 8 | 8 | 6 | 4 | 8 |
|  | TASTPM females | 7 | 7 | 7 |  | 3 | 7 |
| **6 months** | WT males | 6 | 6 | 6 | 5 | 4 | 6 |
|  | WT females | 5 | 5 | 5 |  | 4 | 5 |
|  | TASTPM males | 5 | 5 | 5 | 12 | 3 | 5 |
|  | TASTPM females | 7 | 7 | 7 |  | 3 | 7 |
| **12 months** | WT males | 8 | 8 | 8 | 6 | 3 | 8 |
|  | WT females | 7 | 7 | 7 |  | 3 | 7 |
|  | TASTPM males | 6 | 6 | 6 | 9 | 4 | 6 |
|  | TASTPM females | 8 | 8 | 8 |  | 3 | 8 |
