# Supplementary material for: Neuropathological changes in the TASTPM mouse model of Alzheimer’s disease and their relation to hyperexcitability and cortical spreading depolarization
Source: Sci Rep. 2024 Mar 27;14:7224. doi: 10.1038/s41598-024-57868-4 (PMC10973448; doi:10.1038/s41598-024-57868-4)
Supplement: Supplementary file 3 — Supplementary Table 2. [file 41598_2024_57868_MOESM3_ESM.docx]

Supplementary Table 2

Overview on the numbers of mice used in each group for histological observations. In these animals no electrophysiological recordings were performed.

| **Age** | **Group** | **No. of mice** | **Only Histology** |
| --- | --- | --- | --- |
|  |  |  |  |
| **3 months** | WT males | 3 | 3 |
|  | WT females | 3 | 3 |
|  | TASTPM males | 3 | 3 |
|  | TASTPM females | 3 | 3 |
| **6 months** | WT males | 3 | 3 |
|  | WT females | 3 | 3 |
|  | TASTPM males | 3 | 3 |
|  | TASTPM females | 3 | 3 |
| **12 months** | WT males | 3 | 3 |
|  | WT females | 3 | 3 |
|  | TASTPM males | 3 | 3 |
|  | TASTPM females | 3 | 3 |
